# The Relationship Between Health Literacy Level and Neuropathic Pain Level in Patients With Diabetic Neuropathy

**DOI:** 10.7759/cureus.41490

**Published:** 2023-07-07

**Authors:** Ayşe G Doğan, Ülkem Uzeli

**Affiliations:** 1 Physical Medicine and Rehabilitation, Hitit University Erol Olçok Training and Research Hospital, Çorum, TUR; 2 Internal Medicine, Osmancık State Hospital, Çorum, TUR

**Keywords:** quality of life, neuropathic pain, healty literacy, glycosylated hemoglobin, diabetic neuropahty

## Abstract

Background

This study aimed to analyze the current situation of health literacy (HL), neuropathic pain, and Neuropathic Pain Impact on Quality of Life (NePIQoL) questionnaire in patients with diabetic neuropathy (DN).

Methodology

This study was conducted among 60 patients with diabetic peripheral distal neuropathy on electroneuromyography (ENMG) and 47 patients without diabetic peripheral distal neuropathy on ENMG. The Turkish version of the European Health Literacy Scale (EHLS-TR) for HL levels, Visual Analog Scale (VAS) and Douleur Neuropathique 4 Questions (DN4) for pain level, and NePIQoL for health-related quality of life were used in participants.

Results

A total of 107 type 2 diabetes mellitus patients were included in the study with a mean age of 57.12 ± 4.12 years. The EHLS-TR significantly decreased in the DN group compared to the control group (p = 0.004). There was a significant difference between the two groups in the EHLS-TR classification (p = 0.024). Glycosylated hemoglobin (HbA1c), VAS, and DN4 values were found to be significantly higher in the DN group compared to the control group (p = 0.001). While there was a negative correlation between EHLS-TR scores and DN4 and HbA1c in the DN group, a positive correlation was found between EHLS-TR and NePIQoL.

Conclusions

HL has an effect on HbA1c, neuropathic pain level, and quality of life in DN patients. By increasing the level of HL, glycemic control can be achieved in this patient population, while the level of neuropathic pain decreases and the quality of life increases.

## Introduction

Diabetic neuropathy (DN) is the most common complication of diabetes mellitus which starts distally in the lower extremities and is characterized by loss of sensory function, pain, and severe morbidity [1]. The most common form of DN is distal symmetric polyneuropathy [2]. DN causes an additional burden to the healthcare system due to complications such as foot ulceration, Charcot neuroarthropathy, and lower extremity amputation [3]. The Centers for Disease Control and Prevention defines health literacy (HL) as the degree to which individuals have the ability to find, understand, and use information and services to inform health-related decisions and actions for themselves and others [4]. Insufficient HL in society adversely affects the healthcare system by negatively affecting diagnosis and treatment, causing an increase in hospitalizations and misuse of emergency services [5]. Improving HL in DN patients may enable them to evaluate treatment options and choose the options that are suitable for them, increase their compliance with treatment, and reduce the complications that may occur due to neuropathy by increasing their chances of successful treatment. Although previous studies have demonstrated the relationship between HL and diabetes knowledge, few studies have focused on DN. In this study, we aimed to evaluate the relationship between HL level, neuropathic pain level, and quality of life in patients with DN.

## Materials and methods

The study was approved by the Hitit University Clinical Research Ethics Committee (date: 14.06.2023, decision number: 2023-73). Participants were given detailed information about the study and written consent was obtained in accordance with the Declaration of Helsinki. Patients between the ages of 18 and 75 years who had been diagnosed with diabetes mellitus for at least five years and had undergone glycosylated hemoglobin (HbA1c) testing in the last three months were included in the study. Patients with a history of rheumatic, hypothyroidism, hyperthyroidism, trauma-related extremity surgery, and amputation due to any cause were excluded. This study was conducted among 60 patients with diabetic peripheral distal neuropathy on electroneuromyography (ENMG) and 47 patients without diabetic peripheral distal neuropathy on ENMG who applied to our outpatient clinic. The Turkish version of the European Health Literacy Scale (EHLS-TR) for HL levels, Visual Analog Scale (VAS) and Douleur Neuropathique 4 Questions (DN4) for pain levels, and Neuropathic Pain Impact on Quality of Life (NePIQoL) questionnaire for health-related quality of life were used in participants. EHLS-TR, consisting of 47 items, is used to assess the HL level of individuals over the age of 15. Its reliability and validity have been demonstrated by Okyay et al. in 2016 [6]. The total score ranges between 47 and 88. DN4 is a 10-item scale with a score of 4 or higher defining neuropathic pain [7]. The validity and reliability of Turkish NePIQoL were reported by Acar et al. in 2014 [8]. It includes 42 items in six parameters. The lowest score that can be obtained is 42 and the highest is 210. The score for each item ranges between 1 and 5. An increase in the total score indicates a higher quality of life.

Statistical analysis

SPSS version 22 program (IBM Corp., Armonk, NY, USA) was used for data analysis. The results were evaluated at the 95% confidence interval and the significance level of p < 0.05. Categorical variables were expressed as numbers and percentages. For continuous variables, mean ± standard deviation (SD) or median (minimum-maximum) expressions were used. The Student’s t-test was used to compare HbA1c, VAS, DN4, EHLS-TR, and NePIQoL scores. The chi-square test and Fisher test were used to compare nominal values. The Pearson correlation coefficient was used to determine the relationship among variables.

## Results

A total of 107 diabetic patients, 34 men and 73 women, were included in the study with a mean age of 57.12 ± 4.12 years. While age, gender, marital status, education, and DM duration were similar, job and residential area were different between the groups (p = 0.004). In the DN group, 38 (63.3%) were housewives, 11 (18.3%) were civil servants, and 11 (18.3%) were workers. In the control group, 16 (34.1%) were housewives, 11 (23.4%) were civil servants, and 20 (42.5%) were workers, indicating a significant difference between the groups (p = 0.002) (Table 1). The EHLS-TR significantly decreased in the DN group compared to the control group (p = 0.004). There was a significant difference between the two groups in the EHLS-TR classification (p = 0.024). HbA1c, VAS, and DN4 values were found to be significantly higher in the DN group compared to the control group (p = 0.001) (Table 2). While there was a negative correlation between EHLS-TR scores and DN4 and HbA1c in the DN group, a positive correlation was found between EHLS-TR and NePIQoL (Table 3).

**Table 1:** Demographic characteristics of the DN and control groups. p < 0.05. DN: diabetic neuropathy; SD: standard deviation

	DN group (n = 60)		Control group (n = 47)		P-value
Age (mean ± SD)	58.2±8.8		56.7±9.1		0.246
Gender (F/M)	42/18		31/16		0.124
Married (n)/Single (n)	49/11		38/9		0.358
DM duration (years) (mean± SD)	9.15 ± 2.47		8.24 ± 2.36		0.004
Education	n	%	n	%	0.143
Illiterate	6	10	5	10.6	
Primary school	38	63.3	29	61.7	
Secondary school	15	25	10	21.2	
High school or higher	11	18.3	5	10.6	
Job	n	%	n	%	0.002
Housewife	38	63.3	16	34.1	
Civil servant	11	18.3	11	23.4	
Worker	11	18.3	20	42.5	
Residential area	n	%	n	%	0.004
Village	31	51.6	18	38.3	
District	15	25.0	13	27.7	
City	14	23.3	16	34.0	

**Table 2:** EHLS-TR, DN4, and NePIQoL scores of the DN and control groups. p < 0.05. DN: diabetic neuropathy; SD: standard deviation; DN4: Douleur Neuropathique 4 Questions; VAS: Visual Analog Scale; NePIQoL: Neuropathic Pain Impact on Quality-of-Life Questionnaire; HbA1c: glycosylated hemoglobin; EHLS-TR: Turkish version of European Health Literacy Scale

	DN group (n = 60) (mean ± SD)	Control group (n = 47) (mean ± SD)	P-value
EHLS-TR (score)	26.29 ± 11.09	34.33 ± 9.04	0.034
EHLS-TR classification	n (%)	n (%)	0.029
Insufficient	30 (50.0)	17 (36.1)	
Limited	12 (20.0)	10 (21.2)	
Sufficient	7 (11.6)	11 (23.4)	
Excellent	6 (10)	9 (19.1)	
VAS	7.2 ± 1.23	4.21 ± 1.38	0.001
DN4	6.73 ± 1.21	1.66 ± 1.51	0.001
HbA1c (%)	9.24 ± 1.83	6.37 ± 1.43	0.001
NePIQoL	130.62 ± 27.11		

**Table 3:** Correlation between EHLS-TR score and DN4, HbA1c, VAS, and NePIQoL level in the DN group. DN4: Douleur Neuropathique 4 Questions; VAS: Visual Analog Scale; NePIQoL: Neuropathic Pain Impact on Quality-of-Life Questionnaire; HbA1c: glycosylated hemoglobin; EHLS-TR: Turkish version of European Health Literacy Scale

	EHLS-TR r	EHLS-TR p
HbA1c	-0.477	0.001
DN4	-0.489	0.004
VAS	0.021	0.906
NePIQoL	0.426	0.013

## Discussion

Our results demonstrated that the HL levels were lower in patients with DN. While there was a significant negative correlation between EHLS-TR levels and neuropathic pain and Hb1Ac, there was a positive significant correlation with the quality of life. Diabetes is a group of metabolic diseases characterized by hyperglycemia resulting from defects in insulin secretion, insulin action, or both. The most common complication is neuropathy which can cause foot ulcers, Charcot joints, and amputations [9]. One of the most expensive and debilitating complications of diabetic neuropathy is diabetic foot disease caused by microvascular disease to which prolonged hyperglycemia contributes [10]. The duration of diabetes and HbA1c levels are major predictors of DN [11]. We also found the duration of diabetes and HbA1c levels to be significantly higher in the neuropathy group. If hyperglycemia is controlled, the development and complications of DN can be prevented. This hyperglycemic control was associated with HL level. In a meta-analysis study by Laura et al., higher HL levels were associated with lower HbA1c levels [12]. Gurtoo et al. showed that insufficient HL was an independent predictor of glycemic control and complications [13]. In our study, there was a negative correlation between EHLS-TR and HbA1c. Mohammadi et al. showed that 70% of DM patients without peripheral neuropathy had insufficient HL levels [14]. Among the diabetic peripheral neuropathy patients, the number of HL deficiencies was higher at 74.1% [15]. Regarding HL, diabetic patients with adequate HL were seven times more likely to seek information about diabetes compared to patients with diabetes with limited literacy. Low HL levels also indicate greater susceptibility to disease-related complications [16]. In our results, the level of insufficient HL was significantly higher in the DN group than in the group without neuropathy.

Quality of life is an important factor in DN patients because poor quality of life contributes to decreased self-care, which, in turn, leads to worsened glycemic control, increased risks of complications, and an overwhelming deterioration of diabetes in both the short run and the long run. Yugi et al. reported a positive correlation between HL and quality of life in patients without peripheral neuropathy and in patients with DN [15]. Similarly, many studies have reported that HL both directly and indirectly affects the quality of life positively [17,18]. Because patients with adequate HL have more information about health and the ability to understand and apply this knowledge, they are more likely to make the right health decisions on a daily basis [19,20]. Consistent with the literature, our study revealed that there is a significant positive relationship between HL and quality of life.

As the HL level increases, self-management skills increase and the ability to control the disease becomes stronger. In DN patients, self-management has been increasingly recommended. The HL level should be persistently increased to enable DN patients to gain stronger self-management skills. Compared to DM, it can be said that this patient population needs higher levels of HL as DN patients need more complex self-management strategies [15,21,22]. If self-management skills increase in DN patients, permanent disabilities such as amputation can be prevented and patients’ quality of life can increase [23]. Unfortunately, self-management skills were not addressed in our study. However, the low HL levels in DN patients may indicate that these patients have lower disease management skills than patients without neuropathy.

Limitations

This study has some limitations. First, this study was a cross-sectional study and the number of patients was small. Another limitation was that we did not categorize patients according to neuropathic pain severity and did not compare the quality of life and HL levels between patient groups. Another limitation is that we did not use a diabetic self-management tool. Studies with a larger number of patients and including wider components are necessary to reveal the importance of HL levels in DN patients.

## Conclusions

This study showed that HL has an impact on HbA1c, neuropathic pain level, and quality of life in DN patients. Interventions aimed at enhancing HL would positively improve self-care management skills and patients’ quality of life. With these interventions, the cost to the healthcare system can be reduced by preventing the complications of neuropathy in DM patients. Considering all these findings, we believe that our study will be a reference for more comprehensive studies in DN patients.
